# Influence of Immersion Time on the Frequency Domain Characteristics of Acoustic Emission Signals in Clayey Mineral Rocks

**DOI:** 10.3390/ma17133147

**Published:** 2024-06-27

**Authors:** Jiaju Yan, Zhuxi Li, Dong Xia, Yuxin Bai, Guoliang Shao

**Affiliations:** 1 College of Mining Engineering, North China University of Science and Technology, Tangshan 063210, China; yanjiaju@stu.ncst.edu.cn (J.Y.); lizhuxi@stu.ncst.edu.cn (Z.L.); baiyuxin@ncst.edu.cn (Y.B.); sgl0712@stu.ncst.edu.cn (G.S.); 2 Hebei Province Mining Industry Development with Safe Technology Priority Laboratory, North China University of Science and Technology, Tangshan 063210, China; 3 Hebei Industrial Technology Institute of Mine Ecological Remediation, North China University of Science and Technology, Tangshan 063210, China; 4 Green Intelligent Mining Technology Innovation Center of Hebei Province, Tangshan 063210, China

**Keywords:** immersion time, clay minerals, yellow sandstone, acoustic emission, frequency domain characteristic

## Abstract

The frequency domain characteristics of acoustic emission can reflect issues such as rock structure and stress conditions that are difficult to analyze in time domain parameters. Studying the influence of immersion time on the mechanical properties and acoustic emission frequency domain characteristics of muddy mineral rocks is of great significance for comprehensively analyzing rock changes under water–rock coupling conditions. In this study, uniaxial compression tests and acoustic emission tests were conducted on sandstones containing montmorillonite under dry, saturated, and different immersion time conditions, with a focus on analyzing the effect of immersion time on the dominant frequency of rock acoustic emission. The results indicated that immersion time had varying degrees of influence on compressive strength, the distribution characteristics of dominant acoustic emission frequencies, the frequency range of dominant frequencies, and precursor information of instability failure for sandstones. After initial saturation, the strength of the rock sample decreased from 53.52 MPa in the dry state to 49.51 MPa, and it stabilized after 30 days of immersion. Both dry and initially saturated rock samples exhibited three dominant frequency bands. After different immersion days, a dominant frequency band appeared between 95 kHz and 110 kHz. After 5 days of immersion, the dominant frequency band near 0 kHz gradually disappeared. After 60 days of immersion, the dominant frequency band between 35 kHz and 40 kHz gradually disappeared, and with increasing immersion time, the dominant frequency of the acoustic emission signals increased. During the loading process of dry rock samples, the dominant frequency of acoustic emission signals was mainly concentrated between 0 kHz and 310 kHz, while after saturation, the dominant frequencies were all below 180 kHz. The most significant feature before the rupture of dry rock samples was the frequent occurrence of high frequencies and sudden changes in dominant frequencies. Before rupture, the characteristics of precursor events for initially saturated and immersed samples for 5, 10, and 30 days were the appearance and rapid increase in sudden changes in dominant frequencies, as well as an enlargement of the frequency range of dominant frequencies. After 60 days of immersion, the precursor characteristics of rock sample rupture gradually disappeared, and sudden changes in dominant frequencies frequently occurred at various stages of sample loading, making it difficult to accurately predict the rupture of specimens based on these sudden changes.

## 1. Introduction

Water is one of the most active factors influencing the stability of rock engineering [1,2,3,4]. If rock masses remain in a saturated state for a prolonged period, their strength and stability will continuously decrease, a phenomenon particularly pronounced in rocks containing clay minerals [5,6,7,8]. The information regarding rock loading carried by acoustic emission signals can be reflected in the time domain, spatial domain, and frequency domain characteristics, among which frequency domain features possess uniqueness and intrinsic qualities [9,10,11]. Conducting research on the frequency domain characteristics of acoustic emissions during the process of rupture and instability of clay mineral-containing rocks under the influence of immersion has significant theoretical significance and practical application value for monitoring the stability of surrounding rock under hydro-geological coupling and disaster warning.

During the loading process of rocks, the dominant frequencies of acoustic emission signals are categorized into high and low frequencies based on their frequency. Statistical analysis of acoustic emission dominant frequencies under different loading conditions reveals that high and low frequencies correspond to shear failure and tensile failure during the rock rupture process [12], respectively, with high frequencies corresponding to small-scale cracks and low frequencies to large-scale cracks [13]. Analysis of the acoustic emission dominant frequency characteristics throughout the entire process of uniaxial compression failure of rocks reveals different manifestations of dominant frequencies in various stages of rock failure and deformation [14,15,16,17,18]. In addition to significantly affecting the time-domain parameters of rock acoustic emissions, water also exerts an important influence on frequency-domain parameters. Numerous scholars have conducted related research on the influence of water on the frequency-domain characteristics of rocks [19,20,21]. Based on experimental results regarding the time–frequency characteristics of saturated coal gangue and granite, Zhang et al. [22,23] proposed a new method for predicting rock rupture instability. Zhao et al. [24] conducted acoustic emission experiments on dry, natural, and saturated sandstones under uniaxial compression conditions and found that there was a significant relative decrease in the average acoustic emission energy rate near peak stress. Xu et al. [25] conducted uniaxial compression mechanical and acoustic emission experiments on sandstones with different degrees of saturation and systematically analyzed the b value (a parameter characterizing the relationship between magnitude and frequency of a series of seismic events), RA (the ratio between the rise time of the acoustic emission waveform and the maximum value), and AF values (ratio of the ringing count to the ringing duration), discovering an increase in the proportion of small-scale cracks and shear cracks in saturated rocks. Experimental results by Wang et al. [26] indicated that sandstones in different moisture states exhibit advantages in two frequency bands: 0~75 kHz and 75~150 kHz. An increase in rock moisture content led to a decrease in acoustic emission waveforms within the range of 200~300 kHz and an increase within the range of 0~50 kHz. Duan et al. [27] conducted experimental studies on the time–frequency characteristics of acoustic emissions from water-containing sandstones under uniaxial compression conditions, finding that the drastic fluctuations in time-domain parameters of acoustic emissions indicated that the water-containing sandstone samples were about to fail, providing a theoretical basis for predicting the failure of water-containing rocks in engineering practice. Song et al. [28] conducted Brazilian splitting tensile and uniaxial compression experiments on rocks under different immersion conditions, demonstrating that as the water content increased, the proportion of peak frequency within the range of 0~200 kHz gradually increased. Shan et al. [29] analyzed the influence of water content on the characteristics of infrared radiation and acoustic emission signals during coal failure, finding that water content altered the time–frequency characteristics of coal acoustic emissions. The aforementioned research results provide reliable evidence for the study of precursor information on the failure and instability of water-containing rocks.

For rock engineering projects in hydrogeologically complex conditions or in mines with large water inflows, some rock masses affected by engineering disturbances may remain in a saturated state for a long time. Prolonged saturation not only has a certain deteriorating effect on the mechanical parameters of rocks [30,31,32,33,34] but also significantly affects their acoustic emission parameters [35,36,37,38], especially for rocks containing clay minerals [39,40]. Therefore, the frequency spectrum characteristics of acoustic emissions during the process of rupture and instability of clay mineral-containing rocks under prolonged saturation deserve particular attention.

In this study, sandstones containing clay minerals were selected as experimental samples, and mechanical tests and acoustic emission tests were conducted under uniaxial compression conditions for rock samples subjected to different immersion times. The aim was to explore the influence of immersion time on the frequency spectrum characteristics of acoustic emissions, with a focus on the differences in dominant frequencies of acoustic emissions during the various stress stages of rock rupture and instability under different immersion times. This research expects to provide a reference for the stability evaluation of rock engineering projects under hydrogeologically complex conditions.

## 2. Rock Sample Preparation and Test Method

### 2.1. Rock Sample Preparation

**Figure 1:**
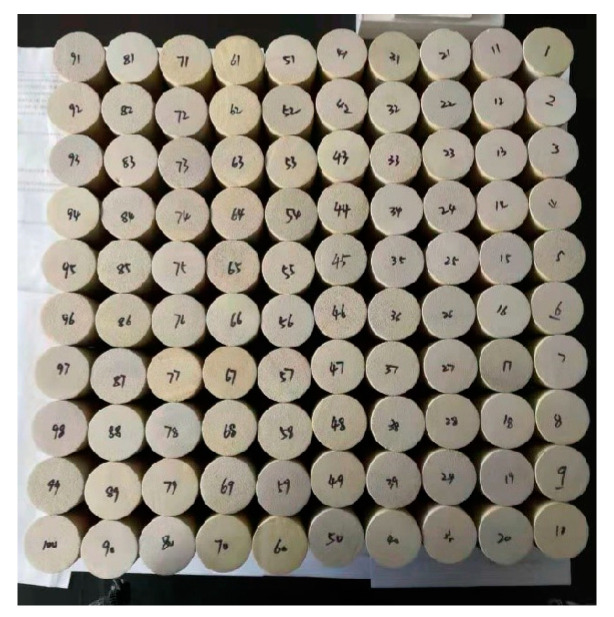
Sandstone samples.

The sample is sandstone, which contains clay minerals. A total of 100 samples were obtained; they were all drilled from completed rock blocks that were taken from a mine site. The specifications of the sample are φ50 × 100 mm according to the requirements of “Rock Test Regulations for Water Conservancy and Hydropower Engineering” (SL264-2020) (the allowable deviation for the height, diameter, or side length of the specimen is ±0.3 mm, and the allowable deviation for the non-parallelism of the two end faces of the specimen is ±0.05 mm). The processed rock samples are shown in Figure 1.

After processing the rock samples, because the rocks have anisotropy, inhomogeneity, and porosity, it is necessary to screen and eliminate the rock samples with obvious defects to ensure the contrast of the test results. After drying the 100 rock samples, the longitudinal wave speed test was conducted by a TICO wave speed tester (TICO, Ridgeland, SC, USA). The abnormal rock samples were removed, and the remaining rock samples were arranged in the order of wave velocity from large to small. Then, the rock samples were grouped. In each group, there were both rock samples with a higher wave speed and a low wave speed, and the rock samples in each group were allocated more consistently to ensure comparable test results. The grouping results are shown in Table 1.

### 2.2. Composition and Structure Analysis of Rock Samples

**Figure 2:**
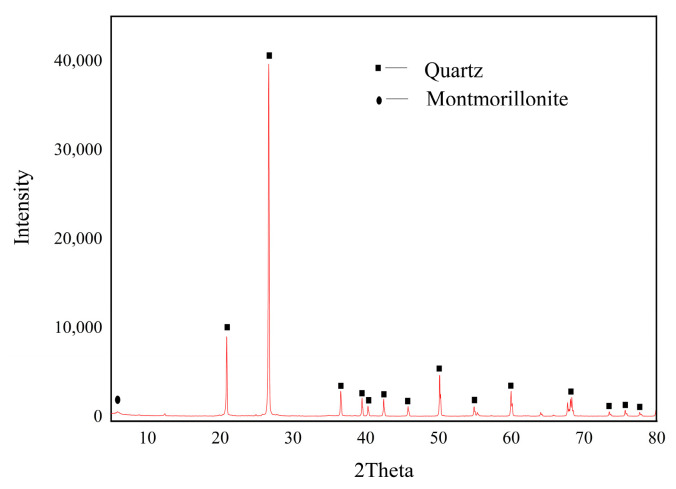
XRD diffraction pattern of yellow sandstone.

After X-ray diffraction analysis (XRD) of rock samples, it is known that the main component of rock is quartz and contains a small amount of clay mineral montmorillonite, among which montmorillonite is a clay mineral. The expansion volume of montmorillonite generated after water absorption can exceed 10~30 times the original volume, which is the main clay mineral component in sandstone. Figure 2 shows the XRD diffraction map.

**Figure 3:**
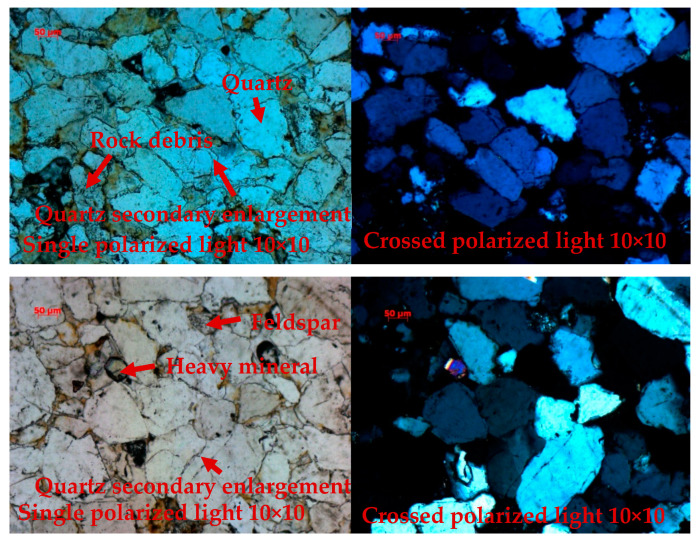
Microstructure of yellow sandstone.

In order to understand the internal structure of the yellow sandstone, the optical microscope OLYMPUS BX53 produced by Olympus Corporation, located in Shinjuku-ku, Tokyo, Japan was used to image and analyze it, as shown in Figure 3.

From the analysis results, it can be seen that the rock samples are mainly fine sand, and the detrital particles are mainly quartz, with a small amount of cuttings and feldspar. All three have dissolution characteristics. Quartz: The particles are mostly secondary and enlarged, with a width of about 5~25 μm; quartz is subcircular–subribess; and the long axis is about 120~250 μm. The short axis is about 50~130 μm. The cuttings are mainly granite and volcaniclastic rock cuttings, subcircular, with a long axis of about 90~180 μm and a minor axis of about 50~90 μm. The cuttings are mainly granite and volcaniclastic rock cuttings, subcircular, with a long axis of about 90~180 μm and a minor axis of about 50~90 μm. Feldspar: mainly potassium feldspar, subcircular, 70~100 μm on the long axis, 45~60 μm on the minor axis. The intergranularity is mainly siliceous, with little clay minerals and occasional heavy minerals. The siliceous is mainly the secondary increase in quartz, which is the first phase of the increase. Clay minerals are cryptocrystalline and mostly attached to the surface of detrital particles.

### 2.3. Test Methods

According to the experimental design, the rock samples were divided into 12 groups, each with 5 rock samples. Group A is a dry rock sample, Group B is a saturated rock sample, and Group C~L is a rock sample soaked for 5, 10, 15, 30, 45, 60, 75, 90, 105, and 120 days, respectively. The free water absorption method was used to saturate the rock samples, and the specimens were immersed in 1/4 every two hours, and the specimens could be immersed in six hours. The natural immersion was maintained for 48 h. The test water was pure water so as to avoid the influence of chemical corrosion in the water–rock interaction caused by the water quality on the test results.

**Figure 4:**
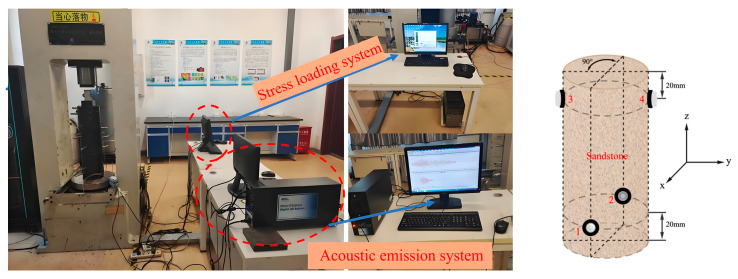
Loading system, AE monitoring system, and processed rock samples.

The loading equipment is the TAW-3000 electro-hydraulic servo conventional triaxial testing machine developed by Changchun Chaoyang Testing Instrument Co., Ltd. in Changchun, China. This machine offers a maximum axial pressure of 3000 kN, a resolution capacity of 20 N, and an axial loading rate ranging from 10 to 10,000 kN/s. The acoustic emission monitoring system utilizes the PCI-2 multi-channel acoustic emission monitoring system produced by Physical Acoustics Corporation (PAC), West Windsor Township, NJ, USA. The selected acoustic emission sensor model R6α is highly sensitive and has a broad application range, meeting all the requirements of this experiment. The rock sample was loaded to 2 kN before the test began, and the acoustic emission sensor was mounted on the fixture. This is shown in Figure 4.

When placing the AE sensor, it is necessary to apply an appropriate amount of petroleum jelly to the contact area between the sensor and the rock sample so that the rock sample and the AE sensor can be better coupled so as to facilitate the transmission of acoustic signals and the synchronous data acquisition of the loading system and the AE monitoring system.

## 3. Effect of Immersion Time on the Mechanical Behavior of Rock Samples

**Figure 5:**
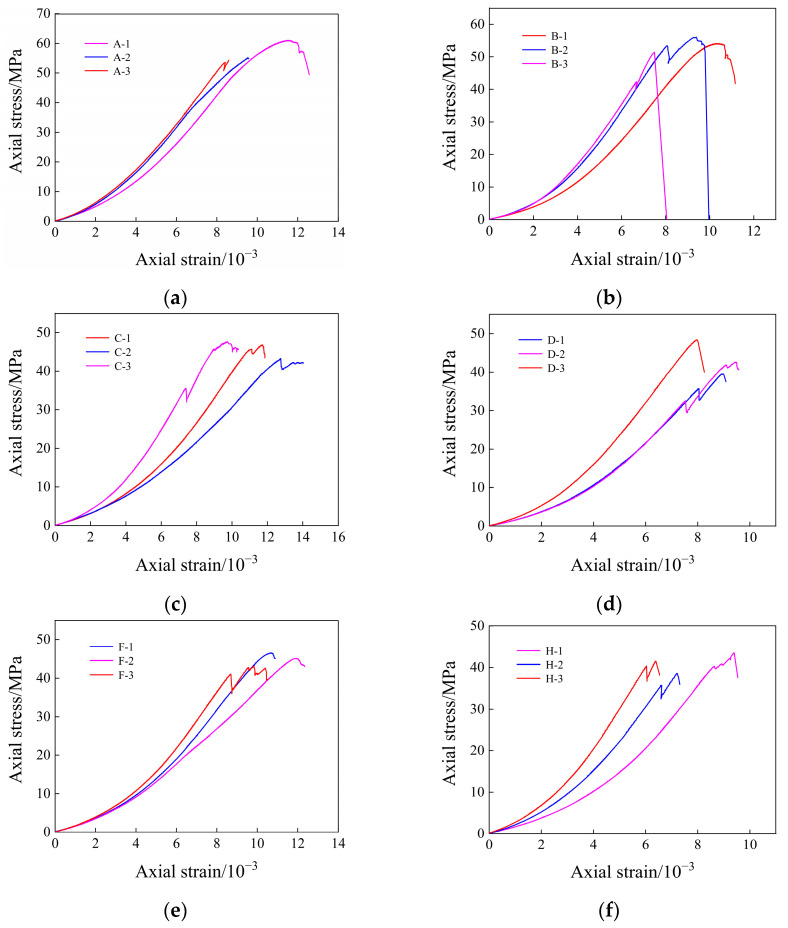

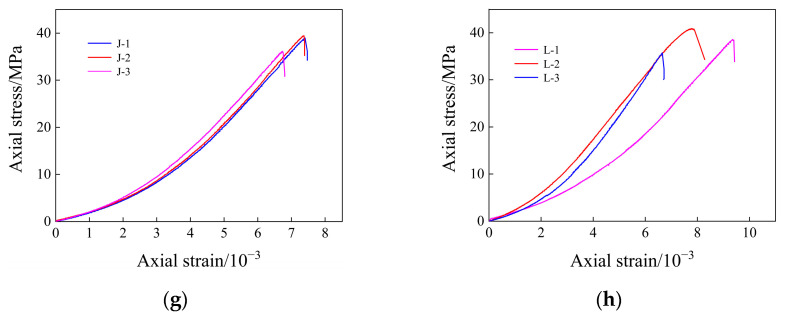
Stress–strain curve of rock at different soaking times: (**a**) dry rock sample; (**b**) water-saturated rock sample; (**c**) immersed in water for 5 days; (**d**) immersed in water for 10 days; (**e**) immersed in water for 30 days; (**f**) immersed in water for 60 days; (**g**) immersed in water for 90 days; (**h**) immersed in water for 120 days.

The stress–strain relationship curve can reflect the damage evolution process of rock samples under load, and according to the test results, the stress–strain relationship curves of dry, saturated, and different immersion times of yellow sandstone are drawn, as shown in Figure 5.

It can be seen from Figure 5 that the immersion time has a certain influence on the deformation characteristics of the saturated rock samples, and the deformation characteristics of each rock sample are relatively close in the compaction stage. After the rock sample is saturated, the clay minerals in it absorb water and swell to promote the fractures [41]. By comparing the stress–strain curves of different immersion times, it can be found that this promotion effect is not significant, indicating that long-term water immersion has little effect on the initial micro-fracture pressure sealing of yellow sandstone. After entering the elastic deformation stage, the energy continues to accumulate in the rock sample, and the comparative analysis of the eight sets of curves in Figure 5 shows that the proportion of the strain corresponding to the elastic deformation increases gradually with the increase in water immersion time. The plastic deformation stage changes most obviously in the four stages of rock fracture and instability, and the amount of plastic deformation gradually decreases with the increase in water immersion time.

The uniaxial compressive strength of dried yellow sandstone is 53.52 MPa, while it decreases to 49.51 MPa after saturation. This is an indication of the softening effect of water. After 5, 10, and 15 days of immersion, the values were 44.93 MP, 43.08 MPa, and 41.2 MPa, respectively, indicating that the immersion time had a continuous weakening effect on the strength of the saturated rock, and the weakening effect was significant. When the immersion time is 30, 45, 60, 75, 90, 105, and 120 days, the intensities are 42.11 MPa, 41.82 MPa, 41.19 MPa, 41.44 MPa, 39.21 MPa, 39.58 MPa, and 39.73 MPa, respectively, which indicates that after the immersion time is more than 30 days, although the rock strength shows a downward trend, the coupling effect of water and rock gradually decreases, and the strength tends to be stable. In the test results, there is a long immersion time and relatively high rock strength. For example, the intensity of immersion in water for 45 d is 0.71 MPa higher than that of immersion in water for 30 d, and the intensity of immersion in water for 75 d is 0.25 MPa higher than that of immersion in water for 60 d, which may be caused by factors such as anisotropy or human operation error in the rock. But overall, the strength of the rock decreases with the increase in immersion time.

## 4. Analysis of the Main Frequency Characteristics of Acoustic Emission Signals

### 4.1. Extraction of the Main Frequency Information of the Acoustic Emission Signal

**Figure 6:**
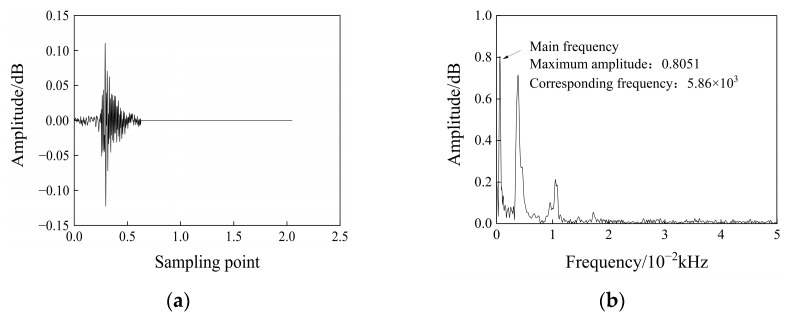
Main frequency extraction method: (**a**) The original acoustic emission signal; (**b**) a two-dimensional spectrogram.

The fast Fourier transform transforms the waveform information of the AE signal into the frequency domain so that it can well represent the global spectral characteristics of the AE signal. The main frequency is the frequency corresponding to the largest amplitude of the two-dimensional spectrogram obtained by the waveform information of the acoustic emission after the Fourier transformation. The programming method is used to convert the acoustic emission waveform data collected in the rock compression process into a batch conversion process, which is converted into a two-dimensional spectrogram that is easy to analyze, and the main frequency parameters are extracted from it. The summary analysis is carried out. The extraction process of the main frequency in the waveform information is shown in Figure 6.

In this paper, the maximum immersion time of the rock sample is 120 days, and uniaxial compressive mechanics and acoustic emission tests are carried out every 15 days. Due to the large amount of AE data and the limitation of space, under the premise of reflecting the test law, the AE main frequency data of dry, saturated, and water-soaked 5 d and 10 d rock samples with large changes in the early stage of the test were analyzed in detail. The coupling effect of water and rock tended to be stable in the later stages of the test. Four groups of rock samples immersed in water for 30 d, 60 d, 90 d, and 120 d were selected for frequency domain characteristic analysis.

### 4.2. Characteristics of the Main Frequency of Acoustic Emission of Dry Rock Samples

**Figure 7:**
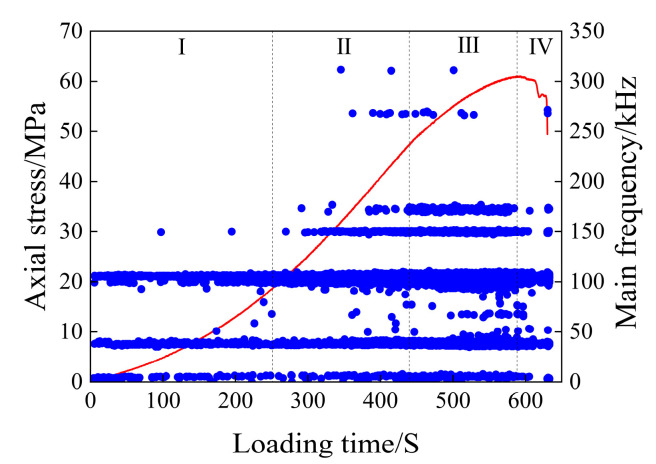
Main frequency scatter diagram of the dry specimen.

The scatter plot of the main frequency of the dry rock sample is shown in Figure 7.

As can be seen from Figure 7, the three main frequency bands of 5 kHz, 45 kHz, and 105 kHz run through the loading process of rock samples. The number of main AE frequencies in the compaction stage (I) is the least compared with the number generated in the whole loading process, which is mainly due to the small number of AE events generated in this stage, which has nothing to do with the variation in the main frequency. The main frequencies at this stage are widely distributed in the three main frequency concentration areas, and only a small number of main frequencies appear between 45 and 105 kHz and about 150 kHz. In the elastic deformation stage (II), the main frequency value between 45 and 105 kHz and about 105 kHz increased, and the main frequency value of about 150 kHz began to appear in large quantities, and the main frequencies of about 170 kHz, 270 kHz, and 310 kHz were added. In the plastic deformation stage (III), the main frequencies of the frequency concentration area of about 45 kHz and 105 kHz increased, the main frequencies of about 150 kHz and 170 kHz began to appear in large numbers, and the main frequencies of about 270 kHz gradually disappeared. After the macroscopic cracks are generated in rock samples (IV), the main frequency of the AE signal is mainly distributed in the three main frequency concentration areas, and only a small amount is distributed in the frequency bands around 150 kHz, 170 kHz, and 270 kHz, and decreases sequentially.

The main frequency of the acoustic emission signal can reflect the magnitude of the fracture propagation in the rock; that is, the high frequency corresponds to the small-scale fracture, and the low frequency corresponds to the large-scale fracture [42]. The main frequency of the AE signal of dry rock samples is 270 kHz and 310 kHz, but the AE signals of this frequency are mainly concentrated in the late stage of elastic deformation and the early stage of plastic deformation of rock loading, which has no research value for the macroscopic failure of rock samples. Therefore, for dry rock samples, the main frequency between 150 and 170 kHz can be defined as high frequency, the main frequency of about 105 kHz is defined as medium and high frequency, the main frequency of about 45 kHz is defined as medium and low frequency, and the main frequency close to 0 kHz is defined as low frequency.

As can be seen from Figure 7, the rock sample generated high-frequency and low-frequency signals during the four loading stages, indicating that the rock sample failure is the result of the gradual accumulation of damage in the rock. It can also be seen from Figure 7 that the compaction stage and elastic stage are mainly compacted large-scale fractures, and there are more small cracks in the later stage of elastic deformation, while the acoustic emission count rate indicates that the number of acoustic emission signals generated is relatively low, suggesting that the development of cracks is minimal and thus has little impact on the overall stability of the rock sample. After the rock sample enters the plastic deformation stage, the high-frequency signal increases and a large number of small-scale fractures begin to occur, which will accelerate the penetration speed of the fractures and eventually lead to the instability and rupture of the rock sample. The number of large and small fractures decreased abruptly after fracture, and the development of small fractures was the main focus, followed by the development of large fractures.

It can be seen that the fracture development rate of the rock sample will increase rapidly before the fracture, and the most significant is the large increase in the high-frequency signal and the abrupt main frequency signal between 45 and 105 kHz, which is the most significant sign before the failure of the rock sample.

### 4.3. Characteristics of the Main Frequency of Acoustic Emission of Saturated Rock Samples

**Figure 8:**
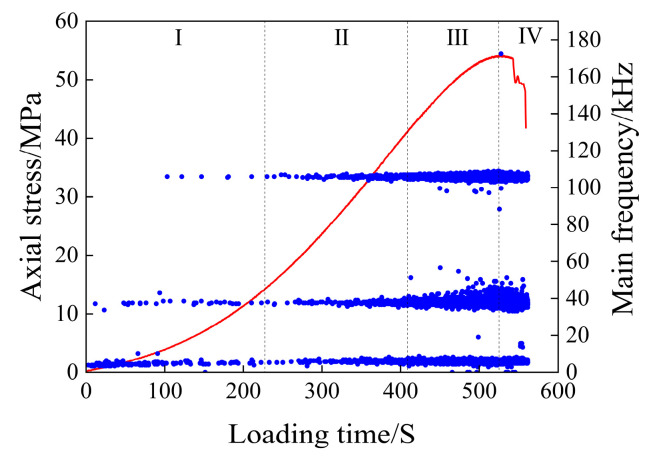
Main frequency scatter diagram of the initial saturated specimen.

The scatter plot of the main frequency of the acoustic emission of the saturated rock sample is shown in Figure 8.

As can be seen from Figure 8, the main frequency of acoustic emission from water-bearing rock samples is mainly concentrated around 5 kHz, 40 kHz, and 105 kHz, which are the three main frequency concentration bands. In the early stage of the compaction stage, the main frequency of the acoustic emission signal is mainly concentrated around 5 kHz, and in the later stage of the compaction stage, the main frequency around 40 kHz and 105 kHz gradually increases and decreases near 5 kHz. In the elastic deformation stage, the acoustic emission signals in the above three main frequency concentration bands showed an increasing trend. In the plastic deformation stage, the acoustic emission signals in the three main frequency concentration bands continued to increase. Among them, the frequency fluctuation range around 40 kHz increases, a wide main frequency band appears, there are multiple mutated main frequencies between 40 and 105 kHz, only a small number of main frequencies appear between 5 and 40 kHz, and a small number of main frequencies close to 0 kHz appear at this time. After entering the instability and rupture stage, the main frequency of the acoustic emission signal is still concentrated around the three frequency bands, and the main frequency of the abrupt signal in the plastic deformation stage basically disappears. There are still a small number of main frequencies close to 0 kHz in this stage.

The main frequency of the saturated rock sample is mainly concentrated at 105 kHz, which can be defined as high frequency, and the frequency tends to be close to 0 kHz, and 5 kHz can be defined as low frequency. In the early stage of loading, there is no high-frequency signal, mainly because the large-scale micro-fractures in the rock sample are preferentially closed during the initial loading, and the small-scale fractures cannot be forced to close due to the small external stress during the initial loading. In the later stage of the micro-fracture compaction stage, with an increase in external load, the number of large-scale fractures decreases, resulting in a decrease in low-frequency signals, and when the small-scale fractures are closed, a small number of high-frequency signals appear in acoustic transmission. During the elastic deformation stage, the acoustic emission signals of the three main frequency bands increased gradually, indicating that the large-scale and small-scale fractures in the rock sample were stably developed. During the elastic deformation stage, the acoustic emission signals of the three main frequency bands increased gradually, indicating that the large-scale and small-scale fractures in the rock sample were stably developed. It is mainly caused by the gradual penetration of adjacent fractures, which makes the fractures develop into an unstable state and finally causes the rock samples to form macroscopic failures. After the macroscopic fracture occurs in the rock, the main frequency signal of the mutation decreases, and the rock enters a stage where the fracture development is relatively stable. A large number of mutant signals appear; the main frequency is relatively concentrated, and the frequency fluctuation range increases.

### 4.4. Characteristics of the Main Frequency of Acoustic Emission of 5-Day Rock Samples Immersed in Water

**Figure 9:**
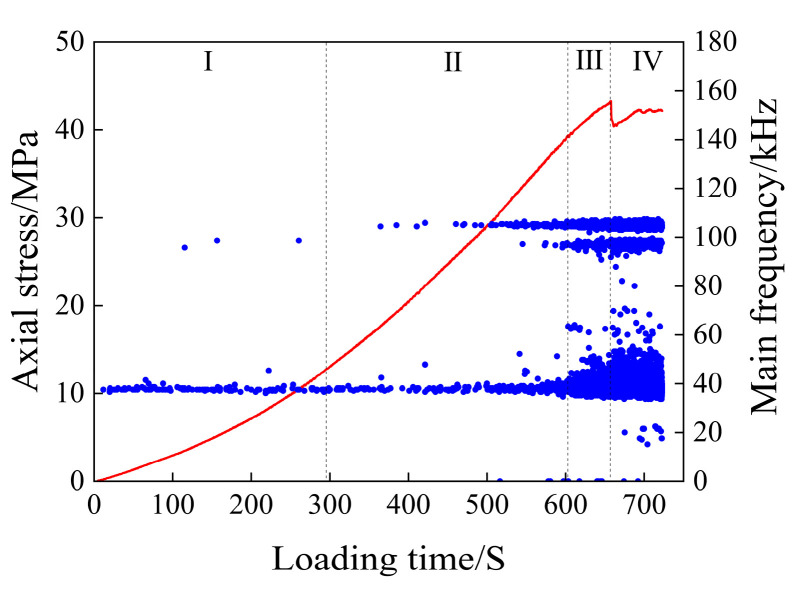
Main frequency scatter diagram of the 5-day immersion test piece.

The scatter plot of the main frequency of the acoustic emission of the rock samples after 5 days of immersion is shown in Figure 9.

As can be seen from Figure 9, the main frequencies of the rock samples after 5 days of immersion are mainly concentrated in the regions around 35 kHz, 95 kHz, and 105 kHz, forming three main frequency bands.

In the compacting stage, the main frequency is concentrated around 35 kHz, and only a small number of them tend to reach 100 kHz. There is no significant mutation of the main frequency, and the main frequency band near 0 kHz completely disappears, resulting in the complete disappearance of signals below the 35 kHz main frequency band. In the elastic deformation stage, the main frequency is around 35 kHz and lasts for the entire deformation stage. In the later stage of the elastic deformation stage, the main frequency of 105 kHz appears and increases with the increase in load. At the end of the elastic deformation stage, the main frequency of 95 kHz appears. In the middle stage of elastic deformation, a small number of mutation signals that are far away from the three main frequency bands appear. In the plastic deformation stage, the acoustic emission signals of the three main frequency bands are generated, and the main frequency band with a frequency of 35 kHz begins to widen; numerous mutation signals emerge between 35 kHz and 70 kHz without forming a distinct frequency concentration band, and the acoustic emission signals approaching 0 kHz start to increase in number.

### 4.5. Characteristics of the Main Frequency of Acoustic Emission of 10-Day Rock Samples Immersed in Water

**Figure 10:**
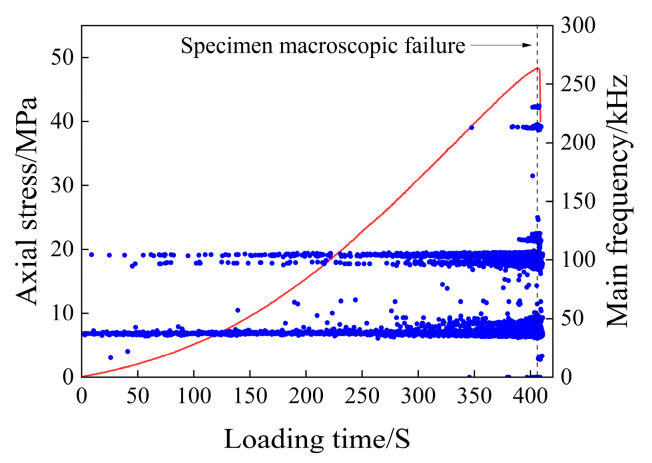
Main frequency scatter diagram of the 10-day immersion test piece.

The scatter plot of the main frequency of the acoustic emission of the rock samples after 10 days of immersion is shown in Figure 10.

As can be seen from Figure 10, the stress–strain curve of the 10-day immersed rock sample is not easy to distinguish between the elastic and plastic deformation stages, so the main frequency scatter plot is no longer divided into failure stages. In order to facilitate the observation of the frequency domain characteristics of the acoustic emission signal before the failure of the rock sample, only the front and back of the macroscopic failure of the rock sample are distinguished, and it is easy to observe the precursor characteristics of the rock sample when the failure occurs. Most of the main frequencies of rock samples are concentrated around 40 kHz, 95 kHz, and 105 kHz, which are the three frequency bands with relatively concentrated main frequencies. In the late loading stage, the main frequency around 95 kHz and 105 kHz fluctuates greatly, and the two frequency bands are close to each other and gradually merge into one main frequency band. In the early stage of loading, a small number of mutant main frequencies are generated, which are distributed between 0 and 40 kHz, and then the main frequency signal between these frequencies disappears. In the middle of loading, more mutant main frequencies began to appear, which were distributed between the two important main frequency bands. With loading, the mutant main frequency gradually increases. In the late loading stage, the main frequency around 40 kHz is broadened to between 40 and 50 kHz. Before the rock sample failure, three main frequency concentration points of 120 kHz, 220 kHz, and 235 kHz were added, and a small number of acoustic emission signals with the main frequency approaching 0 kHz appeared.

After 10 days of immersion, the duration of the plastic deformation stage of the saturated rock sample becomes shorter, and it is not easy to divide, but the change characteristics of the rock sample before fracture can still be directly observed from the main frequency scatter map. As can be seen from Figure 10, the variation characteristics of the main frequency before the rupture of the specimen are as follows: Both high-frequency and low-frequency signals show an increasing trend, and the frequency width of the main frequency band increases. A large number of mutant main frequencies between the two main frequency bands appear. Ultra-low and ultra-high frequency signals begin to be generated.

### 4.6. Characteristics of the Main Frequency of Acoustic Emission of Long-Term Flooded Rock Samples

According to the test results, the scatter plots of the main frequency of acoustic emission during the instability and rupture of rock samples with water immersion times of 30 days, 60 days, 90 days, and 120 days were drawn to analyze the influence of long-term water immersion on the precursors of rock fracture.

**Figure 11:**
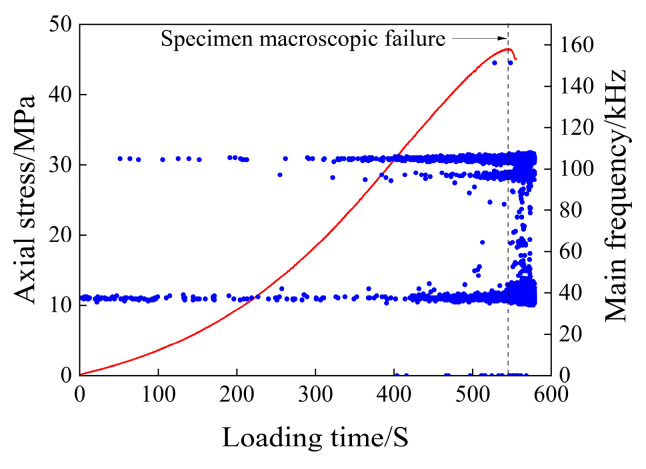
Main frequency scatter diagram of the 30-day immersion test piece.

The scatter plot of the main frequency of the acoustic emission of the rock samples after 30 days of immersion is shown in Figure 11.

As can be seen from Figure 11, after 30 days of water immersion, the main frequencies are mainly concentrated around 40 kHz, 95 kHz, and 105 kHz, which are the three main frequency bands. The abrupt main frequency far away from the main frequency band is mainly distributed between 40 and 95 kHz in the two main frequency bands, and only a small amount is distributed around 0 kHz and 155 kHz. It can be seen that the frequency variation characteristics of rock samples immersed in water for 30 days before fracture are as follows: The frequency fluctuation range of the two main frequency bands increases, and the abrupt main frequency away from the main frequency band begins to appear and gradually increases. Among them, the occurrence and large increase in the main frequency of abrupt changes are the most significant precursor characteristics before the fracture of rock samples.

**Figure 12:**
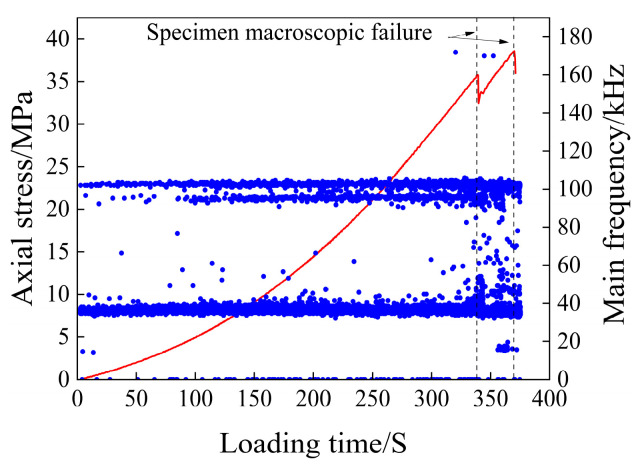
Main frequency scatter diagram of the 60-day immersion test piece.

The scatter plot of the main frequency of the acoustic emission of the rock samples after 60 days of immersion is shown in Figure 12.

It can be seen from Figure 12 that after 60 days of water immersion, the main frequency of the rock sample is mainly concentrated around 35 kHz, 95 kHz, and 105 kHz. The abrupt main frequency is mainly around 0 kHz and between 35 and 95 kHz, which is distributed throughout the whole process of rock sample loading. The abrupt main frequency, with a frequency between 0 and 35 kHz, appeared in small quantities at the beginning of loading and after the first macroscopic failure of the rock sample. The abrupt main frequency of around 170 kHz only appears for a short period of time before and after the first macroscopic failure of the rock sample.

Affected by its own defects, after the rock sample was immersed in water for 60 days, there were two macroscopic failures during the loading process. After the first failure, the fracture was small, and the rock sample did not lose its bearing capacity. After 50 s of continuous loading, the second failure occurred in the rock sample, and the interval between the two times was short. Therefore, the changes in the main frequency after the first failure and before the second failure can be regarded as precursor characteristics of rock fracture. The precursor characteristics of the first failure of rock samples are as follows: the abrupt main frequency begins to appear near 170 kHz, the abrupt main frequency of 0 kHz begins to continue to occur, and the number of abrupt main frequencies away from the main frequency band between 35 and 95 kHz increases slightly. The precursor characteristics of the second failure of rock samples are as follows: The abrupt main frequency between 0 and 35 kHz begins to appear, and the number is large. A large number of mutant main frequencies between 35 and 95 kHz are generated. There is also a slight increase in the abrupt frequency around 170 kHz.

**Figure 13:**
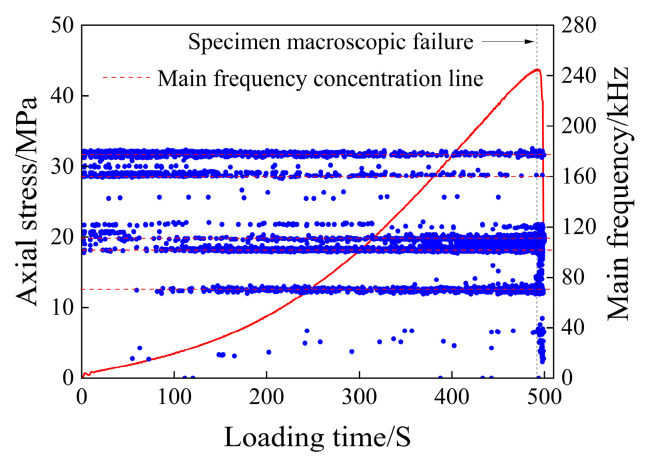
Main frequency scatter diagram of the 90-day immersion test piece.

The scatter plot of the main frequency of the acoustic emission of the rock samples after 90 days of immersion is shown in Figure 13.

As can be seen from Figure 13, after 90 days of water immersion, the main frequency is scattered, and the frequencies that obviously form the main frequency band are mainly distributed around 70 kHz, 100 kHz, 110 kHz, 160 kHz, and 180 kHz, and a large number of mutant main frequencies away from the main frequency band are generated between 0 kHz and 180 kHz. The main changes in the main frequency of the specimen before the destruction are as follows: the high-frequency signals near 160 kHz and 180 kHz in the main frequency band gradually decrease, the number of main frequencies of the acoustic emission signal near 70 kHz, 100 kHz, and 110 kHz in the main frequency band gradually increases, and the number of abrupt main frequency signals far away from the main frequency band increases slightly. In general, the precursor characteristics of the specimens before rupture after 90 days of immersion were not significant.

**Figure 14:**
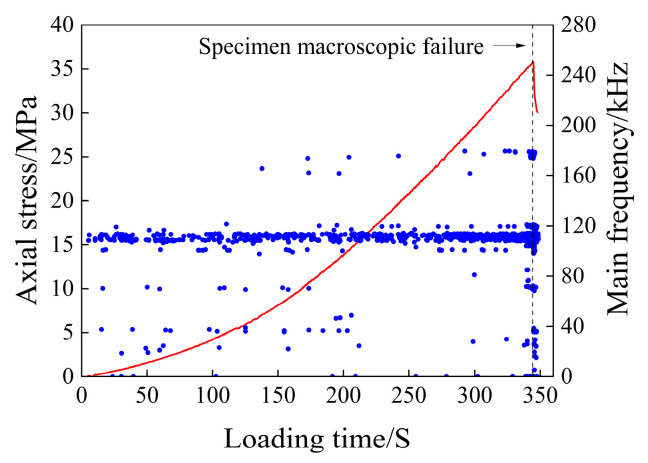
Main frequency scatter diagram of the 120-day immersion test piece.

The scatter plot of the main frequency of the acoustic emission of the rock samples after 120 days of immersion is shown in Figure 14.

After 120 days of immersion, the number of acoustic emission signals generated during the loading process of the rock sample is significantly reduced, so the number of main frequencies is also reduced accordingly. As can be seen from Figure 14, the main frequency is concentrated around 110 kHz, forming a dominant frequency band, and there are a small number of scattered main frequency signals between 0 and 110 kHz. In the middle and late stages of loading, the main frequency signal is also generated near 180 kHz, but the number is small and cannot form a more concentrated main frequency band. Throughout the whole process of rock sample loading, it can be seen that the main frequency of the rock sample does not change significantly before failure. In a short period of time before the rock sample failure, a large number of main frequency signals began to be generated near 35 kHz, 70 kHz, and 180 kHz, but the duration was short, and the early warning significance of the specimen rupture was not significant.

Based on the above analysis, it can be seen that the variation in the main frequency of the acoustic emission signal during the loading process of long-term flooded rock samples is not significant. In the early stage of loading, the main frequency is scattered and scattered, and the abrupt main frequency signals far away from the main frequency band also begin to appear in large numbers in the early stage of loading, which has a great impact on the prediction of rock fracture. When the rock samples were immersed in water for 90 days and 120 days, the precursor characteristics of the failure basically disappeared.

### 4.7. A Comprehensive Analysis of the Influence of Immersion Time on the Main Frequency Characteristics of Acoustic Emission Signals

Through the analysis of the main frequency characteristics of the AE signal, it can be seen that the main frequency of the AE signal generated during the loading of rock samples is concentrated, which will gather in a certain frequency band to form a dominant frequency band, and a small number of abrupt main frequency signals will also be generated in the area far away from the main frequency band. Experimental results show that long-term immersion reduces the number of acoustic emission signals, and consequently, the quantity of main-frequency signals of acoustic emissions also decreases. Table 2 presents the relationship between immersion time and the main frequency parameters of acoustic emissions.

As can be seen from Table 2, the number of acoustic emission signals in dry rock samples is the largest. The number of saturated rock samples was 52.6% of that of dry rock samples. The main frequencies of saturated 5 d, 10 d, 30 d, 60 d, 90 d, and 120 d rock samples were 49.1%, 20.1%, 19.8%, 21.4%, 16.9%, and 2.8% of those of dry rock samples, respectively. According to the conclusion that the main frequency can reflect the size of the fracture development scale, it can be seen that the rock sample will produce fractures of the same size at the same time point during the loading process. Comparing the scatter plots with similar numbers of four main frequencies for 10~90 d, it can be seen that with the increase in soaking time, the scale of fracture development in rock samples during the loading process is different, and fractures of multiple scales develop together. The length of immersion time leads to a change in the frequency domain characteristics of the acoustic emission signal during the loading process of the rock sample. Since the three main frequency bands of 95 kHz, 105 kHz, and 110 kHz are similar, they can be approximated as one main frequency band, and a main frequency band will be generated between 95 and 110 kHz at different immersion times, which can be regarded as the most important frequency band. After the rock sample was immersed in water for 5 days, the main frequency band near 0 kHz gradually disappeared, and the low-frequency signal approaching 0 kHz was not easy to generate. When the rock sample was immersed in water for 60 days, the main frequency band between 35 and 40 kHz gradually disappeared. With the increase in immersion time, the main frequency band of low frequency gradually disappears, and the main frequency of the acoustic emission signal increases, indicating that long-term immersion will lead to a decreasing trend in the development scale of rock fractures.

The immersion time also has an important influence on the frequency range of the main frequency of the rock sample acoustic emission signal. During the loading process of dry rock samples, the main frequency of the acoustic emission signal is mainly concentrated in the range of 0~310 kHz, and only a small number of signals are generated near 310 kHz. After the rock sample is waterlogged, the high-frequency signal near 310 kHz disappears. The high-frequency signal is around 230 kHz and appears only in small quantities when immersed in water for 10 days. The main frequency of the rest of the immersion time is below 180 kHz. The influence of long-term immersion on the frequency range of rock acoustic emission signals is reflected in the fact that the number of main frequencies of low frequencies is greatly reduced, and the frequency of high frequencies is reduced.

The main frequency of the acoustic emission signal can predict the instability and fracture of rock samples at different immersion times, and the most significant feature of dry rock samples before rupture is the occurrence of a large number of high-frequency and abrupt main frequencies. The precursor characteristics of rock fracture in 5 d, 10 d, and 30 d of saturated and flooded rock samples are the appearance and rapid increase in abrupt main frequencies and an increase in the main frequency range. After 60 days of immersion, the precursor characteristics of rock sample rupture gradually disappeared, and the abrupt main frequency frequently appeared in all stages of rock sample loading. The abrupt main frequency could not accurately predict the rupture of the specimen.

## 5. Analysis of the Characteristics of the Main Frequency Signal of Acoustic Transmission

The test results show that the immersion time has a significant effect on the acoustic emission frequency domain parameters of the clay-bearing mineral yellow sandstone. Because the rock contains quartz chips, a small amount of rock chips, and feldspar chips, all three of which have the characteristics of dissolution. At the same time, the inside of the rock also contains clay minerals such as montmorillonite, which is easy to expand when exposed to water, which makes the internal microstructure of the rock easy to change after encountering water. The characteristics of the AE signal are determined by the development of the internal fractures in the rock, and the influence of water on the characteristic parameters of the AE signal is caused by the interaction of water and rock on the development and expansion of the micro-fractures in the rock. Through the summary analysis, the influence of water on the acoustic emission signal of rock can be summarized into four points:

(1) After water enters the rock through the original micro-fractures of the rock, it can lead to the dissolution of quartz debris, feldspar debris, and other materials with dissolution characteristics in the rock, resulting in the destruction of the internal structure of the rock and then the weakening of the rock’s strength and the further development of fractures.
(2) The presence of water will reduce the cohesion between the particles inside the rock, so that the initial defects of the rock will be magnified. During the rock loading process, the development of fractures tends to concentrate on the defect parts, and the development and expansion rate of the cracks in the defects are slow, which changes the development law of the fractures during drying.
(3) Water will also fill the original micro-fractures in the rock, and some of the micro-fractures will heal and close when the rock sample is pressurized, and the water will produce additional stress in the closed fractures. The stress will act on the periphery of the rock pores, which will change the stress state of the rock when it is dry, causing the damage of the larger defective parts first and thus changing the development and expansion of the fractures.
(4) The water content of the rock leads to the softening and mudding of the argillaceous mineral components, which, together with the water, play a shock-absorbing role, resulting in the acceleration of the weakening speed of the acoustic signal inside the rock. With an increase in immersion time, this effect becomes more obvious, so that the small signal cannot be transmitted to the surface of the rock sample to be received by the instrument.

## 6. Conclusions

The frequency domain characteristics of acoustic emission reflect that rock structure and stress conditions are difficult to analyze in time domain parameters. Studying the influence of immersion time on the mechanical properties and acoustic emission frequency domain characteristics of clay mineral rocks is of great significance for comprehensively analyzing rock changes under water–rock coupling conditions.

The uniaxial compression and acoustic emission tests were conducted on yellow sandstone under dry, saturated, and saturation durations of 5, 10, 30, 45, 60, 75, 90, 105, and 120 days. The effects of immersion time on the frequency domain signal characteristics of rock acoustic emissions were analyzed, and the conclusions are as follows:

(1) The uniaxial compressive strength of dried yellow sandstone is 53.52 MPa, while it decreases to 49.51 MPa after saturation. This is an indication of the softening effect of water. The strengths corresponding to immersion durations of 5, 10, 15, 30, 45, 60, 75, 90, 105, and 120 days were 44.93 MPa, 43.08 MPa, 41.20 MPa, 42.11 MPa, 41.82 MPa, 41.19 MPa, 41.44 MPa, 39.21 MPa, 39.58 MPa, and 39.73 MPa, respectively. This indicates that water significantly weakens the strength of rock samples in the early stages of immersion. After 30 days of immersion, the coupling effect between water and rock gradually decreases, and the strength of the samples stabilizes.
(2) Immersion time has a significant impact on the distribution characteristics of the dominant frequencies of acoustic emission signals in mudstone. Dry and initially saturated rock samples exhibited three dominant frequency bands. However, different immersion durations resulted in a dominant frequency band appearing between 95 kHz and 110 kHz. After 5 days of immersion, the dominant frequency band near 0 kHz gradually disappeared, making it difficult for low-frequency signals near 0 kHz to occur. After 60 days of immersion, the dominant frequency band between 35 kHz and 40 kHz gradually disappeared. With increasing immersion time, low-frequency dominant bands gradually disappeared, and the dominant frequencies of acoustic emission signals showed an increasing trend.
(3) Immersion time also affects the frequency range of dominant frequencies in rock acoustic emission signals. During the loading process of dry rock samples, the dominant frequency of acoustic emission signals is mainly concentrated between 0 kHz and 310 kHz, with few signals near 310 kHz. After saturation, the high-frequency signals near 310 kHz disappeared, and high-frequency signals appeared near 230 kHz, with only a small amount occurring after 10 days of immersion, while the dominant frequencies for the remaining immersion durations were all below 180 kHz. The long-term immersion affects the frequency range of dominant frequencies in rock acoustic emission signals by significantly reducing the number of low-frequency dominant frequencies and lowering the frequencies of high-frequency signals.
(4) The frequency-domain characteristics of acoustic emission signals can be used to predict the instability and rupture of rock samples under different immersion durations. For dry rock samples, the most significant features before rupture are the frequent occurrences of high-frequency signals and numerous sudden changes in the dominant frequencies. For initially saturated samples and those immersed for 5, 10, and 30 days, the precursor characteristics include the frequent appearance and rapid increase in these sudden changes in dominant frequencies, as well as an expansion in the frequency range of these dominant frequencies. However, after 60 days of immersion, these precursor characteristics gradually disappear. Additionally, sudden changes in dominant frequencies frequently occur at various stages of sample loading, making it difficult to accurately predict the rupture of specimens based on such changes at this stage.

## Figures and Tables

**Table 1:** Wave Velocity (V) of Rock Samples in Each Group.

No.	V (m/s)	No.	V (m/s)	No.	V (m/s)	No.	V (m/s)	No.	V (m/s)	No.	V (m/s)
A-1	2023	C-1	2030	E-1	2087	G-1	2000	I-1	2013	K-1	2107
A-2	2140	C-2	2153	E-2	2203	G-2	2130	I-2	2137	K-2	2210
A-3	2250	C-3	2253	E-3	2277	G-3	2223	I-3	2240	K-3	2307
A-4	2343	C-4	2350	E-4	2383	G-4	2323	I-4	2330	K-4	2397
A-5	2443	C-5	2453	E-5	2483	G-5	2430	I-5	2437	K-5	2490
B-1	2030	D-1	2133	F-1	2107	H-1	2010	J-1	2017	L-1	2130
B-2	2147	D-2	2210	F-2	2207	H-2	2137	J-2	2140	L-2	2220
B-3	2250	D-3	2317	F-3	2290	H-3	2230	J-3	2247	L-3	2320
B-4	2343	D-4	2400	F-4	2390	H-4	2327	J-4	2330	L-4	2427
B-5	2447	D-5	2503	F-5	2483	H-5	2437	J-5	2443	L-5	2523

**Table 2:** Main frequency parameters of different soaking times.

Soaking Time/d	Number of Main Frequencies	Main Frequency Band/kHz	Frequency Range/kHz
Dry	81,697	0, 45, 105	0~310
Saturated	42,951	5, 40, 105	0~180
5	40,109	35, 95, 105	0~110
10	17,133	40, 95, 105	0~230
30	16,171	40, 95, 105	0~110
60	17,516	35, 95, 105	0~170
90	13,876	70, 100, 110, 160, 180	0~180
120	2286	110	0~180

## Data Availability

The original contributions presented in the study are included in the article, further inquiries can be directed to the corresponding author.
